# *N*-Methylcytisine Ameliorates Dextran-Sulfate-Sodium-Induced Colitis in Mice by Inhibiting the Inflammatory Response

**DOI:** 10.3390/molecules23030510

**Published:** 2018-02-25

**Authors:** Yan-Fang Jiao, Min Lu, Yu-Ping Zhao, Ning Liu, Ya-Ting Niu, Yang Niu, Ru Zhou, Jian-Qiang Yu

**Affiliations:** 1 Department of Pharmacology, Ningxia Medical University, Yinchuan 750004, China; 18709600923@163.com (Y.-F.J.); yaolilumin@163.com (M.L.); nxsfxyzyp@163.com (Y.-P.Z.); liuning11@163.com (N.L.); Niuyating@163.com (Y.-T.N.); 2 Key Laboratory of Hui Ethnic Medicine Modernization, Ministry of Education, Ningxia Medical University, Yinchuan 750004, China; niuyang0227@163.com; 3 Ningxia Hui Medicine Modern Engineering Research Center, Ningxia Medical University, Yinchuan 750004, China

**Keywords:** inflammatory bowel disease, *N*-methylcytisine, anti-inflammatory activity, pro-inflammatory cytokines, nuclear factor-kappa B (NF-κB)

## Abstract

This study aimed to investigate the anti-inflammatory effects of *N*-methylcytisine (NMC) in a dextran sulfate sodium (DSS)-induced colitis model and explore its possible mechanisms. Experimental colitis was induced by administering the mice with 5% DSS for 7 days. Different doses of NMC (1, 4 and 16 mg/kg) and 5-aminosalicylic acid (100 mg/kg) were given orally once every day for 7 days. The protective effect of NMC was evaluated using the disease activity index, colon length and results of histopathological examination. The possible mechanisms of NMC were explored by evaluating the expression levels of tumour necrosis factor-α, interleukin-1β and interleukin-6 (IL-6) using ELISA and analysing the protein expression levels of nuclear factor (NF)-κB p65, p-NF-κB p65, p-IκB, IκB, IκB kinase (IKK) and p-IKK using western blots. Results demonstrated that the oral administration of NMC attenuated the DSS-induced clinical symptoms and pathological damage. In addition, NMC treatment significantly reduced myeloperoxidase activity and level of pro-inflammatory cytokines. Further studies revealed that NMC blocked the activation of NF-κB by inhibiting IκB and IKK phosphorylation. These findings suggested that NMC exerts anti-inflammatory effects on DSS-induced colitis, and its mechanism may be related to the suppression of NF-κB activation. Thus, NMC may have potential therapeutic value in the treatment of colitis.

## 1. Introduction

Inflammatory bowel disease (IBD), most commonly referring to ulcerative colitis (UC) and Crohn’s disease (CD), is a complex, multifactorial, immune mediated gastrointestinal disorder characterised by chronic relapsing inflammation in the gut [1]. Several reports indicated that the risk of colorectal cancer development in patients with IBD gradually increases [2]. The prevalence and incidence of IBD have been persistently increasing worldwide over the past few decades [3]. IBD greatly affects the health and quality of life of millions of people; thus, it is considered a global health issue.

However, the aetiology of IBD remains unclear. Many studies have implicated that different environment factors, genetic susceptibility, intestinal microbiota and immune responses are involved in the development of IBD [4]. IBD results from an uncontrolled mucosal immune response to intestinal microflora in genetically susceptible hosts. The dysfunction of the immune response produces large amounts of pro-inflammatory cytokines (tumour necrosis factor-α [TNF-α], interleukin-1β [IL-1β] and interleukin-6 [IL-6]), reactive oxygen species, nitric oxide and prostaglandins in the colonic mucosa [5,6]. The transcription factor nuclear factor (NF)-κB, which regulates the expression of inflammatory mediators and serves as a core transcription factor in immune and inflammatory reactions, amplifies the inflammatory cascade and ultimately causes colonic tissue damage [7,8]. Hence, the overproduction of inflammatory mediators and excessive activation of NF-κB are considered important processes in the pathogenesis of IBD.

Establishing a suitable animal model of colitis is imperative to elucidate its underlying patho-physiologic mechanisms and assess the potency of therapeutic agents. Numerous chemical-induced animal models of colonic inflammation have been widely used to investigate the disease [9,10,11,12]. The oral administration of dextran sulfate sodium (DSS) has been the most commonly employed method of inducing colitis, and such a model closely resembles clinical IBD in terms of the pathogenesis of inflammation, pro-inflammatory cytokines and morphological features. This model also exhibits relative simplicity and high reproducibility compared with other models of colitis [13,14,15]. Hence, the DSS-induced colitis model is especially suitable in studying the mechanism of inflammatory colitis and identifying potential therapeutic compounds for colitis.

Currently, the most common drugs used for the treatment of colitis include 5-aminosalicylates, mesalamine, corticosteroids, immunosuppressants and anti-TNF-ɑ monoclonal antibodies, which suppress the intestinal inflammatory burden and improve the disease-related symptoms. However, most drugs cause multiple adverse effects [16]. As a result, searching for safer and more effective agents of treatment for colitis remains a critical issue.

**Figure 1:**
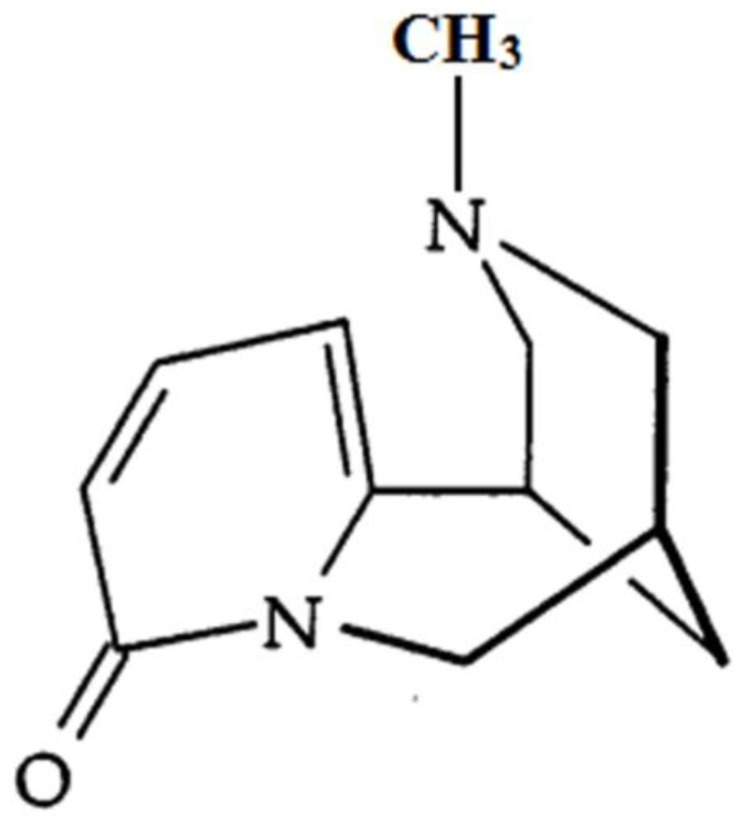
The chemical structure of *N*-methylcytisine (NMC).

*N*-Methylcytisine (NMC; Figure 1), a tricyclic quinolizidine alkaloid extracted from the seeds of *Laburnum anagyroides* Medik and *Sophora alopecuroides* L., exerts a variety of pharmacological activities, such as hypoglycaemic, analgesic and anti-inflammatory activities [17,18]. However, the protective effects of NMC on DSS-induced colitis have not been previously explored. The purpose of this study, therefore, is to determine the anti-inflammatory activity of NMC on DSS-induced colitis in mice and to explore its possible mechanisms.

## 2. Results

### 2.1. NMC Ameliorated the Clinical Symptoms of DSS-Induced Colitis in Mice

**Figure 2:**
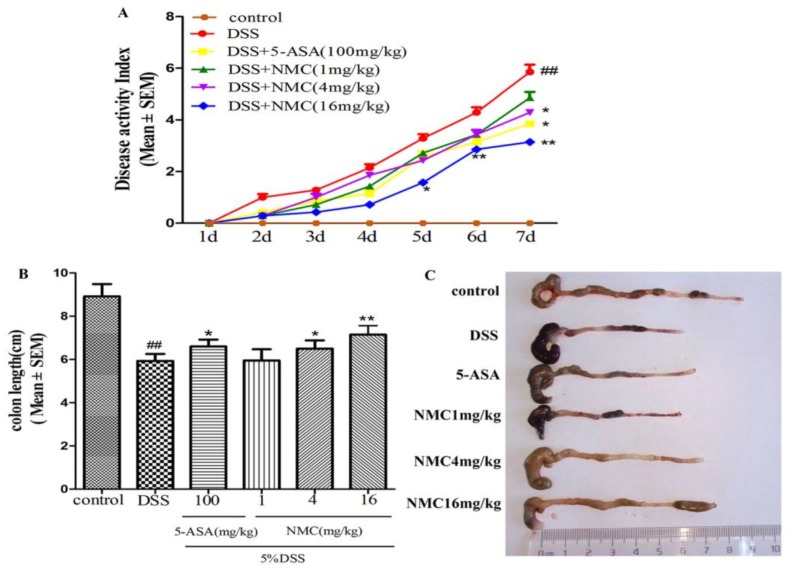
NMC ameliorates clinical signs of DSS-induced acute murine colitis. (**A**) Effects of different doses of NMC and 5-ASA on DAI in DSS-induced experimental colitis in mice. (**B**) Effects of NMC on DSS-induced changes in colon length and (**C**) macroscopic appearances. Data are expressed as mean (*n* = 10 per group) ± SEM. ## *p* < 0.01 compared with control group; * *p* < 0.05, ** *p* < 0.01 compared with the DSS-vehicle group.

The mice treated with 5% DSS for 7 days developed acute colitis, and they exhibited many phenotypic features of relevance to human ulcerative colitis [19]. The disease activity index (DAI) score, as a credible marker of colonic inflammation associated with weight loss, diarrhoea and rectal bleeding, was obtained. As shown in Figure 2A, the DAI score of the DSS-treated group was significantly elevated compared with that of the control mice (*p* < 0.01). The DAI scores of the NMC (4 and 16 mg/kg) and 5-aminosalicylic acid (5-ASA, 100 mg/kg) treatment groups were markedly reduced compared with the DAI score of the mice treated with DSS alone. However, NMC treatment at 1 mg/kg did not attenuate the DSS-mediated increase in DAI scores.

The colon length also indirectly indicated the severity of DSS-induced colitis. DSS typically causes colonic shortening. Such a change was evidently improved by the administration of 4 and 16 mg/kg NMC and 100 mg/kg 5-ASA (Figure 2B,C). However, no significant difference was observed after NMC treatment (1 mg/kg; *p* > 0.05).

### 2.2. NMC Attenuated the DSS-Induced Colonic Histopathological Changes

**Figure 3:**
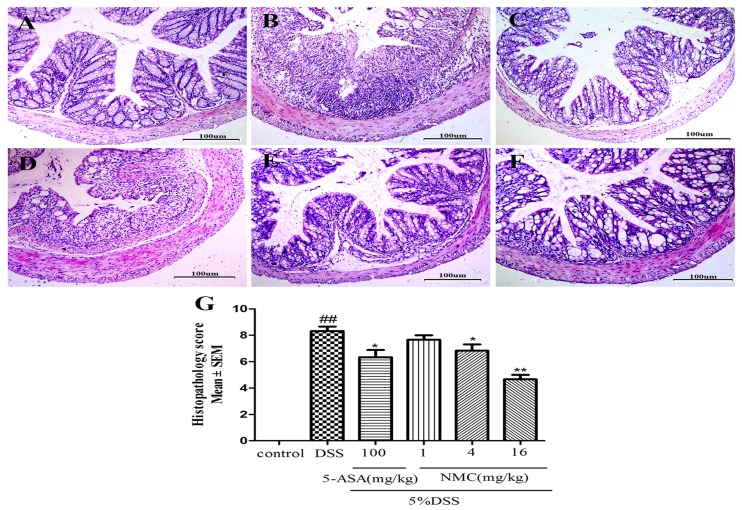
Effects of NMC on histological changes of colonic tissue in DSS-induced mouse colitis. Section were stained with hematoxylin and eosin: (**A**) control; (**B**) DSS; (**C**) 5-ASA (100 mg/kg); (**D**) NMC (1 mg/kg); (**E**) NMC (4 mg/kg); (**F**) NMC (16 mg/kg); (**G**) histological scores. Data are expressed as mean (*n* = 10 per group) ± SEM. ## *p* < 0.01 compared with control group; * *p* < 0.05, ** *p* < 0.01 compared with the DSS-vehicle group.

We determined the severity of colonic inflammation and ulceration via haematoxylin and eosin (H&E) staining. The histological examination is shown in Figure 3. The colons of the control group mice exhibited normal integrated colonic architecture and morphology of crypts and abundant goblet cells (Figure 3A, Table 1). However, the DSS-treated group presented severe epithelial damage, crypts loss, depletion of the goblet cells and abundant inflammatory cell infiltration mainly in the mucosa and submucosa (Figure 3B, Table 1). The oral administration of MNC (4 and 16 mg/kg) and 5-ASA reduced the extent of colon injury, congestion, oedema and inflammatory cells in comparison with the DSS-only group (Figure 3C,E,F, Table 1). The low-dose NMC (1 mg/kg) did not show a significant difference (Figure 3D, Table 1). The histopathological score corresponded with the result of HE staining (Figure 3G). Severe inflammation-induced damage exhibited in the DSS-induced colitis group was ameliorated with high doses of NMC (4 and 16 mg/kg).

### 2.3. NMC Decreased the Activity of Myeloperoxidase (MPO) in the Colon 

**Figure 4:**
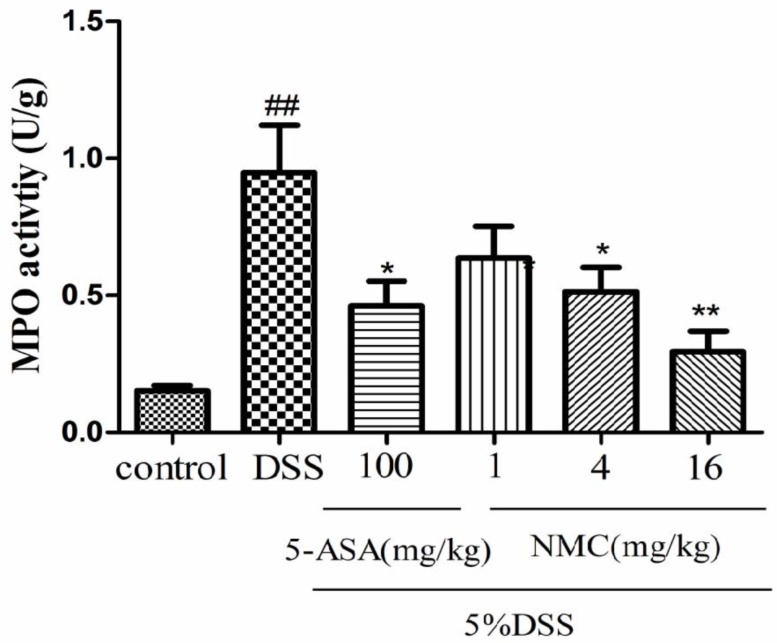
Effects of NMC on the MPO activity of colonic tissue from mice with DSS-induced colitis. Data are expressed as mean (*n* = 10 per group) ± SEM. ## *p* < 0.01 compared with control group; * *p* < 0.05, ** *p* < 0.01 compared with the DSS-vehicle group.

MPO is an enzyme present in neutrophils and at a much lower concentration inmonocytes and macrophages. The level of MPO activity can positively reflect the number of neutrophils [20,21]. As indicated in Figure 4, MPO activity of the mice with DSS-induced colitis significantly increased compared with that of the untreated DSS mice (*p* < 0.01). The administration of different doses of NMC (4 and 16 mg/kg) evidently reduced MPO activity in comparison with the administration of DSS. This finding was consistent with the result of 5-ASA treatment. Nevertheless, no statistically significant differences were observed between the NMC (1 mg/kg) and DSS groups (*p* > 0.05).

### 2.4. NMC Reduced the Pro-Inflammatory Cytokines in the Colon

**Figure 5:**
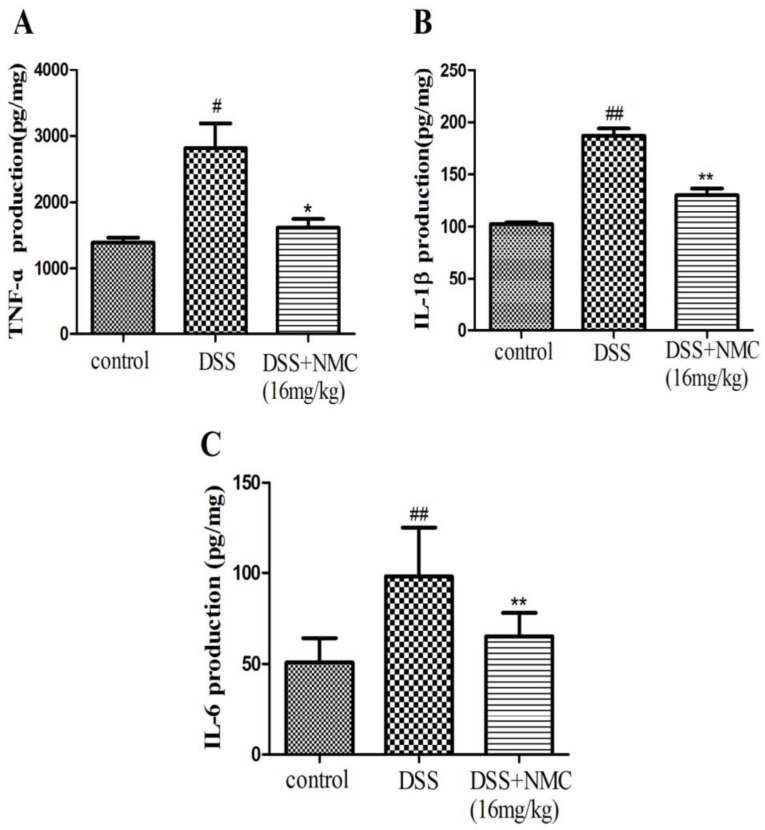
Effects of NMC on levels of TNF-α, IL-6 and IL-1β of colon in DSS-induced colitis using ELISA. (**A**) TNF-α, (**B**) IL-1β, (**C**) IL-6. Data are expressed as mean (*n* = 10 per group) ± SEM. # *p* < 0.5, ## *p* < 0.01 compared with control group; * *p* < 0.05, ** *p* < 0.01 compared with the DSS-vehicle group.

Pro-inflammatory cytokines, such as TNF-α, IL-1β and IL-6, play important roles in colitis development. To test whether blocking these pro-inflammatory cytokines can ameliorate colitis in mice, we examined the levels of TNF-α, IL-1β and IL-6 in the inflamed colonic tissue using ELISA. The mucosal concentration of the cytokines increased in the DSS-induced group compared with the control. However, the oral administration of NMC (16 mg/kg) significantly reduced the concentrations of TNF-α, IL-1β and IL-6 in the colonic tissue compared with the DSS-induced mice and markedly down-regulated the expression of the cytokines (Figure 5).

### 2.5. NMC Suppressed the Expression of p-NF-κB p65, IκB and IκB Kinase (IKK) in DSS-Induced Colitis

**Figure 6:**
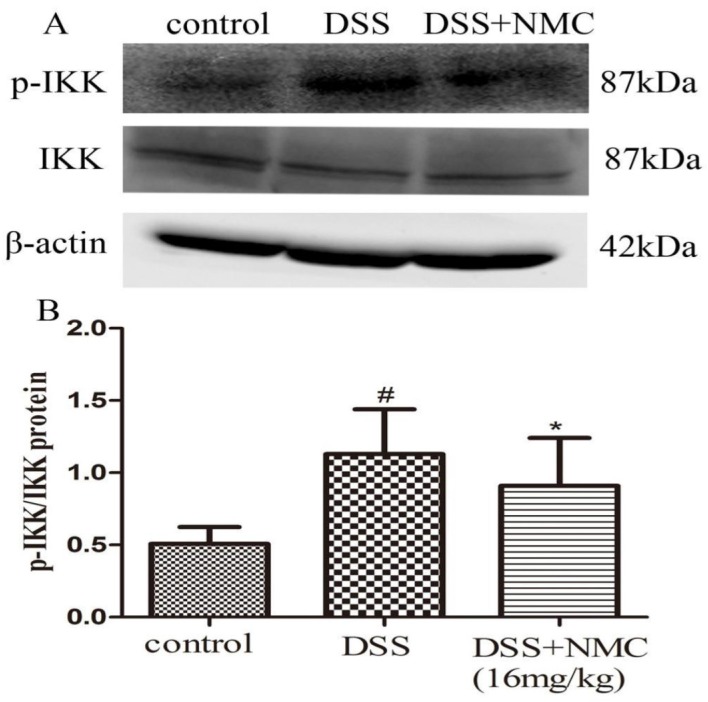
Effects of NMC on the expression of p-IKK and IKK in DSS-induced colitis. (**A**) The expression of p-IKK and IKK in colon tissue were determined using Western blotting. (**B**) The relative quantification of p-IKK and IKK protein expression calculated by using Image J software. Data are expressed as mean (*n* = 10 per group) ± SEM. # *p* < 0.5 compared with control group; * *p* < 0.05 compared with the DSS-vehicle group.

**Figure 7:**
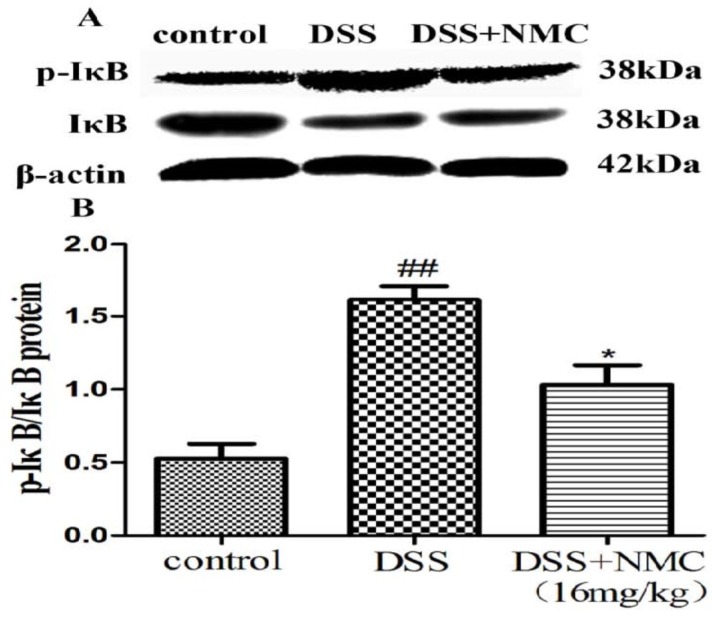
Effects of NMC on the expression of p-IkB and IkB in DSS-induced colitis. (**A**) The expression of p-IkB and IkB in colon tissue were determined using Western blotting. (**B**) The relative quantification of p-IkB and IkB protein expression calculated by using Image J software. Data are expressed as mean (*n* = 10 per group) ± SEM. ## *p* < 0.01 compared with control group; * *p* < 0.05 compared with the DSS-vehicle group.

**Figure 8:**
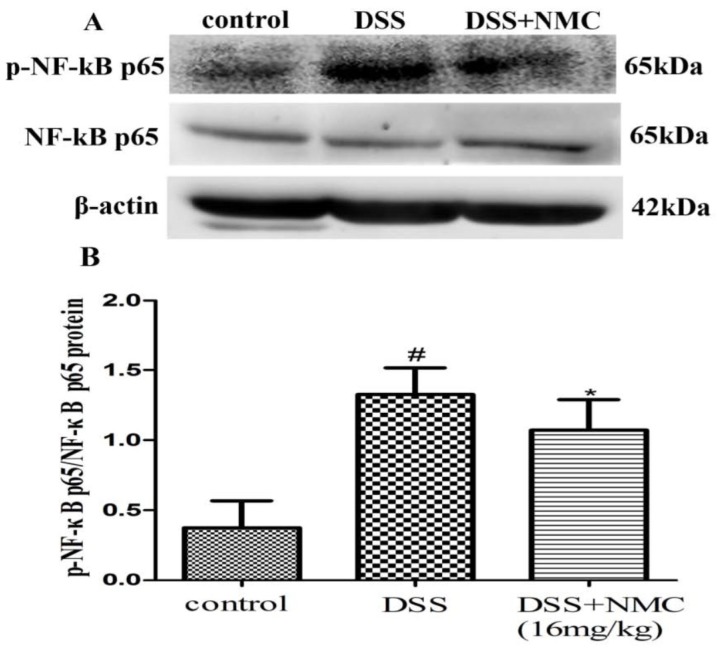
Effects of NMC on the expression of p-NF-kB p65 and NF-kB p65 in DSS-induced colitis. (**A**) The expression of p-NF-kB p65 and NF-kB p65 in colon tissue were determined using Western blotting. (**B**) The relative quantification of p-NF-kB p65 and NF-kB p65 protein expression calculated by using Image J software. Data are expressed as mean (*n* = 10 per group) ± SEM. # *p* < 0.05 compared with control group; * *p* < 0.05 compared with the DSS-vehicle group.

NF-κB, a pivotal transcription factor, regulates the expression of pro-inflammatory genes, which plays an important role in the progression of IBD [22]. Western blot was employed to further determine whether the anti-inflammatory effect of NMC on the expression of pro-inflammatory mediators is correlated with the blockade of NF-κB activation in colonic tissues. We found that the protein expression of IκB decreased but that of p-NF-κB p65, p-IκB and p-IKK increased in the DSS-induced group compared with the control. NMC treatment significantly increased IκB expression, decreased the expression of IκB and IKK phosphorylation and inhibited p-NF-κB p65 expression (Figure 6, Figure 7 and Figure 8).

## 3. Discussion

IBD is considered a chronic autoimmune disorder with an unclear aetiology. In general, the aberrant intestinal inflammatory response is thought to contribute to IBD development [1,23]. Numerous therapeutic agents targeting specific components of the immune response have been shown to be effective in controlling the symptoms. Unfortunately, these drugs have potential side effects, including steroid dependence and serious infections [24,25,26]. Therefore, novel therapeutic candidates, especially natural drugs, with high efficacy and safety are urgently required. Our present study demonstrated for the first time that the therapeutic effects of NMC on DSS-induced colitis suppressed inflammation. 

The administration of DSS dissolved in drinking water to mice for several days induces a colitis that is characterised by hematochezia, body weight loss and shortening of the intestine. Moreover, the microscopic morphological features of the colon show evident damages, including crypt loss, mucosal ulceration and infiltration with neutrophil granulocytes [11,27]. The findings of our present study agreed with previous reports. The mice treated with 5% DSS in drinking water for 7 days presented similar clinical symptoms. NMC administration during colitis induction by DSS significantly ameliorated the inflammatory signs, such as colon length, body weight loss and colonic tissue damage. This observation indicated the protective effect of NMC on DSS-induced colitis by ameliorating body weight loss, shortening of the intestine and pro-inflammatory cytokine infiltration. 

Substantial evidence showed that the infiltration of inflammatory cells, including neutrophils and macrophages, is a hallmark of the disease pathophysiology of IBD [28]. MPO is a peroxidase enzyme that is abundantly expressed in neutrophils and is a biomarker of gut acute inflammation and oxidative stress in experimental IBD [29]. Furthermore, MPO is involved in the generation of hypochlorous acid and tyrosyl radicals, these compounds possess strong antibacterial and antiviral properties. However, free radicals, such as reactive oxygen and nitrogen species, can damage body cells, leading to the destruction of proteins, DNA and lipids. Eventually, such destruction causes colonic tissue damage and mucosal dysfunction, which play a significant role in the pathogenesis of IBD [30]. The level of MPO activity adequately reflects the degree of tissue infiltration by neutrophils. In this study, our data showed that colonic MPO activity significantly increased in the DSS group. NMC effectively suppressed MPO activity after 7 days of treatment. These observations confirmed that the beneficial influence of NMC in colitis was correlated with a decrease in local inflammation.

Accumulating literature has revealed that pro-inflammatory cytokines play a key role in the pathogenesis of IBD [6,31]. These cytokinese, such as TNF-ɑ, IL-1β and IL-6, are mainly produced by immunocompetent cells, which mediate inflammation initiation and propagation and facilitate the interaction between cells [32]. The overexpression of TNF-α is critical in intestinal mucosal lesions [33]. TNF-α is a pleiotropic cytokine produced by intestinal epithelial cells and macrophages; it induces the activation of other cells and promotes chemokine expression and secretion of other cytokines, particularly adhesion molecules. Therefore, TNF-α is an indispensable mediator of pathological inflammation in IBD. IL-6 is a pro-inflammatory cytokine produced by various cell types and exerts pleiotropic effects. IL-1β is a critical cytokine that modulates immune cells and is correlated with the severity of inflammation [34,35,36]. Our present study showed that the induction of colitis by DSS led to damage and inflammatory infiltration of the colonic mucosa and increased the mucosal concentration of TNF-α, IL-1β and IL-6. By contrast, NMC administration decreased the mucosal concentration of the pro-inflammatory cytokines in mice. These data further indicated that NMC exhibited anti-inflammatory properties by reducing the local inflammatory response in DSS-induced colitis. This effect was possibly one of the mechanisms involved in the NMC-evoked protective effect in the colon.

An interesting discovery in our present study concerned the inhibitory effect of NMC on the activation of NF-κB in DSS-induced colitis. With the development of molecular medicine, studies on nuclear transcription factors, such as NF-κB, have garnered increasing attention. NF-κB controls several important physiological, immune and inflammatory responses. Pro-inflammatory cytokines, such as TNF-α and IL-1β, interact with the NF-κB signalling pathway. These triggering substances activate the NF-κB signalling pathway, which in turn promotes the expression of the inflammatory mediators, including TNF-α, IL-1β and IL-6 [37]. Therefore, the activation of NF-κB is a critical step in the activation and propagation of inflammatory responses in human IBD and animal colitis [20]. Moreover, numerous clinical studies suggested that the application of NF-κB p65 oligonucleotides significantly decreases the expression of NF-κB p65 and cytokines in the mucosa and ameliorates the severity of clinical symptoms [38]. Normally, NF-κB, as a heterodimer complex of (p50/p65), is regulated by IκB and IKK. IKK resides in the cytoplasm in an inactive state and is bound to the inhibitory protein IκB. The activation of the IKK complex is accelerated under harmful stimulating factors. Subsequently, IKKs phosphorylate the inhibitory IκBα protein. NF-kB becomes activated, translocates into the nucleus, binds target DNA elements and encodes a variety of inflammatory mediators [39,40,41]. Our present study showed an increased expression of p-IKK and p-IkB and the translocation of p-NF-κB p65 in the DSS-induced mice; this finding was consistent with the results of other reports. NMC administration suppressed IκB degradation, IKK phosphorylation and p-NF-κB p65 expression in the colonic tissues of the DSS-treated mice. These results indicated that the anti-inflammatory effects of NMC on DSS-induced colitis could be associated with the blockade of cytokine-mediated NF-κB activation.

In summary, the oral administration of NMC, an alkaloid obtained from natural plants, could ameliorate DSS-induced colitis in mice by inhibiting the up-regulation of pro-inflammatory cytokines and NF-κB activation. Therefore, NMC may be a promising protective agent recommended for IBD patients. However, these findings suggested that the anti-inflammatory effect of NMC on DSS-induced colitis could be related to NF-κB. Further studies are warranted to elucidate the underlying mechanisms and intensively evaluate the therapeutic potential of NMC in colitis.

## 4. Materials and Methods

### 4.1. Drugs and Reagents

DSS (36–50 kDa) was purchased from MP Biochemicals, LLC (Solon, OH, USA). NMC (purity > 98%, ZhongKeZhiJian Biotechnology Co., Ltd., Beijing, China) was dissolved in normal saline (NS). 5-ASA (Sigma–Aldrich, St. Louis, MO, USA) was dissolved in 0.05% sodium carboxymethyl cellulose solution. MPO kits were purchased from Jiancheng Bioengineering Institute (Nanjing, China). ELISA kits for TNF-α, IL-1β and IL-6 were purchased from RayBiotech, Inc. (Atlanta, GA, USA). All other reagents were of analytical grade and sourced from standard commercial suppliers. 

### 4.2. Animals

Male ICR mice weighing 20 ± 2 g were purchased from the Experimental Animal Centre of Ningxia Medical University (certificate number: SYXK Ningxia 2005-0001). The animals were acclimatised for 3 days prior to the experiment and maintained in the animal house with 23 °C ± 2 °C ambient temperature and 12 h light–dark cycles. They had access to food pellets and water ad libitum during the whole experiment. All the experimental protocols were approved by the institutional animal ethics committee of Ningxia Medical University (Yinchuan, Ningxia, China).

### 4.3. Induction of Colitis and Experimental Design 

Colitis was induced by administering the mice with 5% DSS dissolved in drinking water for 7 days. The animals were randomly divided into six groups (*n* = 10 mice/group), namely, the control, DSS (only), DSS + 5 − ASA (100 mg/kg), DSS+NMC (1 mg/kg), DSS+NMC (4 mg/kg) and DSS+NMC (16 mg/kg) groups. Normal and colitic controls were administered with NS by oral gavage. 5-ASA and NMC were given intragastrically once per day in an application volume of 0.1 mL/10 g body weight from day 1 to day 7. The body weight of the mice was measured and recorded every day during the experimental period.

### 4.4. Assessment of Disease Severity

The daily clinical symptoms of DSS-induced colitis in mice were evaluated. DAI reflected the intestinal disease activity and was calculated by scoring the weight loss, diarrhoea and rectal bleeding following the method previously described by Cooper [42,43] (Table 2). The DAI values were the sum of the weight loss, stool consistency and faecal occult blood scores. All groups of mice were sacrificed on day 8. The colons were removed, and the length was measured. The excised colon was washed thoroughly with ice-cold PBS. The distal small section of the colon was cut and fixed in 10% neutral buffered formalin for histological examination, and the remaining portion was immediately frozen at −80 °C for further analysis.

### 4.5. Histopathological Evaluation of Colon

The fixed distal colonic tissue samples were embedded in paraffin and sectioned at 4 μm thick. All the tissue sections were stained with H&E for the evaluation of colonic histopathological changes under light microscopy (Olympus, Tokyo, Japan) at 100× magnification. Each H&E-stained section was scored by a pathologist who was blinded to the interventions. The severity of colitis was assessed in accordance with a previously described scoring system [44]. In brief, the assessment included the loss of crypts, extent of injury and mucosal damage and inflammatory cell infiltration. The histological score was as follows: the inflammation severity was scored on a 0–3 scale (0: none; 1: slight; 2: moderate; 3: severe) as was the extent of injury (0: none; 1: mucosal; 2: mucosal and submucosal; 3: transmural); crypt damage was scored on a 0–4 scale (0: none; 1: basal third damaged; 2: basal two-thirds damaged; 3: only surface intact; 4: loss of entire crypt and epithelium); and the percentage of the colon involved was scored on a 0–4 scale (0: none; 1: 1–25%; 2: 26–50%; 3: 51–75%; 4: >75%). One slide per sample was evaluated for the histological score. The score ranged from 0 to 14 (total score) and represented the sum of the scores for severity and extent of inflammation, crypt damage and percentage of the colon involved.

### 4.6. Myeloperoxidase (MPO) Activity Assay

MPO is an important marker of neutrophil influx into colonic tissues. The proteins extracted from the colonic tissues were used to assess the MPO levels in accordance with the manufacturer’s instructions. The excised colon was weighed, homogenised in 0.1 M phosphate buffer (pH 7.4) and centrifuged. The supernatant was used to determine the MPO concentration. Absorbance was measured at 460 nm. MPO activity was represented as U/g protein and defined as the quantity of enzyme degrading 1 μmol peroxide per minute at 37 °C.

### 4.7. Cytokine Analysis by ELISA

Frozen colonic tissues were homogenised with lysis buffer to extract the total protein. The homogenate was centrifuged at 12,000 ×*g* at 4 °C for 5 min. The supernatant obtained was used for the quantification of the levels of TNF-α, IL-1β and IL-6 using commercially available ELISA kits (TNF-α, IL-1β and IL-6, Ray Biotech, Inc.) according to the manufacturer’s instruction. The amount of total protein concentration in the supernatant was determined using the BCA protein assay kit (Jiangsu KeyGen Biotech Co., Ltd., Nanjing, China).

### 4.8. Western Blot Analysis

The colonic tissues from each group of mice were homogenised with lysis buffer containing a protease inhibitor, phosphatase inhibitor and 100 mM PMSF (Jiangsu KeyGen Biotech Co., Ltd.) and then centrifuged at 12,000 ×*g* at 4 °C for 5 min. The protein concentration of the remaining supernatant was determined using the BCA protein assay kit. Equal amounts of protein (50–70 µg) were separated using 10% SDS–polyacrylamide gel electrophoresis and electrotransferred onto a sheet of nitrocellulose membrane. The membrane was blocked with 5% non-fat milk in PBS with Tween 20 (PBST) for 2 h at room temperature. The blocking membrane was incubated with the corresponding primary antibodies of p-NF-κB p65 (1:500, Cell Signalling Technology, Danvers, MA, USA), NF-κB p65 (1:1000, Cell Signalling Technology), IκB (1:500, Proteintech Group, Chicago, IL, USA), IKK (1:200, Proteintech Group), p-IκB (1:500, SAB, College Park, MD, USA), p-IKK (1:500, SAB, USA) and β-actin (1:2000, Elabscience, Shanghai, China) at 4 °C overnight. β-Actin was used as the housekeeping protein for the whole tissue extracts. Subsequently, the membrane was washed three times for 10 min each with PBST and incubated with the specific secondary antibodies (goat anti-rabbit, 1:2000, Proteintech Group) for 2 h. Protein bands were visualised using a luminol-based enhanced chemiluminescence (Advansta, Menlo Park, CA, USA) reagent. The images were quantified via densitometry with a western blot detection system (Quantity One software; Bio-Rad Laboratories, Hercules, CA, USA).

### 4.9. Statistical Analysis

Results were expressed as mean ± SEM. One-way ANOVA and the Student–Newman–Keuls test were employed for post hoc comparisons to determine the differences between the control and experimental groups. Student’s t-test was performed for paired samples. Statistical significance was set at *p* < 0.05.

## Figures and Tables

**Table 1:** Effect of NMC on morphological signs of colonic damage observed seven days in DSS-induced colitis.

Morphological Changes				
Groups	Inflammation Severity (0–3)	Injury Extent (0–3)	Crypt Damage (0–4)	Involved Colon (0–4)
**Control**	0	0	0	0
**DSS**	2–3	3	3	2
**5-ASA+DSS**	2	1–2	2	1
**NMC(1 mg/kg)+DSS**	3	2–3	3	2
**NMC(4 mg/kg)+DSS**	1–2	2	2	1
**NMC(16 mg/kg)+DSS**	1	2	1	1

Numbers represent the predominant histological grading in each group.

**Table 2:** Criteria for disease activity index.

Score	Weight Loss (%)	Stool Consistency	Occult/Gross Bleeding
0 1 2 3 4	None 1–5 5–10 10–20 >20	Normal Loose stool Diarrhea	Negative Positive Gross bleeding

DAI = (stool consistency) + (gross bleeding) + (weight loss) was used to evaluate the severity of intestinal inflammation in clinic.
